# Mechanisms of Omega-3 Polyunsaturated Fatty Acids in Prostate Cancer Prevention

**DOI:** 10.1155/2013/824563

**Published:** 2013-05-23

**Authors:** Zhennan Gu, Janel Suburu, Haiqin Chen, Yong Q. Chen

**Affiliations:** ^1^State Key Laboratory of Food Science and Technology, School of Food Science and Technology, Jiangnan University, Wuxi 214122, China; ^2^Department of Cancer Biology, Wake Forest University School of Medicine, Medical Center Boulevard, Winston-Salem, NC 27157, USA

## Abstract

This review focuses on several key areas where progress has been made recently to highlight the role of omega-3 polyunsaturated fatty acid in prostate cancer prevention.

## 1. Introduction

The health benefits of omega-3 polyunsaturated fatty acids (n-3 PUFA), mainly eicosapentaenoic acid (EPA 20:5) and docosahexaenoic acid (DHA, 22:6), have been long known. Epidemiologic studies dating back to the 1970s were among the first to suggest that dietary PUFA may be beneficial in preventing disease [1, 2]. Still today, studies continue to demonstrate the health benefits of n-3 PUFA; however, the mechanisms of action of n-3 PUFA are still not fully understood. Many new discoveries have advanced our understanding about the activities of n-3 PUFA against human disease. For example, DHA-receptor GPR120 has been demonstrated to play a role in sensing and controlling obesity and metabolic syndrome [3]; the recently identified omega-3 mediators, resolvins, and protectins have been demonstrated to have anti-inflammatory and proresolving activities [4]. The purpose of this review is to highlight the recent advances in our understanding of the mechanisms by which n-3 PUFA modulate prostate cancer development.

## 2. Fatty Acids

 There are two major classes of PUFA: n-6 and n-3. Unlike saturated and monounsaturated fatty acid, PUFA cannot be synthesized *de novo* by mammals because they lack the required enzymes and, therefore, PUFA must be obtained from the diet. The n-6 and n-3 PUFA also cannot be interconverted in mammals, but within each series, their metabolism can produce various lipids that differ in chain length and number of double bonds. Linoleic acid (LA, 18:2 n-6) is an n-6 PUFA found in high concentration in grains as well as many seeds and meats. LA serves as a substrate to be converted into a longer fatty acid, arachidonic acid (AA, 20:4 n-6), via a series of oxidative desaturation and elongation reactions. Of the n-3 fatty acids, alpha linolenic acid (ALA, 18:3 n-3) is found at moderate levels in plants, seeds, leafy vegetables, legumes, and nuts. ALA is not metabolized efficiently to longer-chain n-3 PUFA, such as EPA and DHA.

 Although they belong to two distinct families, n-3 and n-6 PUFA are metabolized by some of the same enzymes, specifically, delta-5-desaturase and delta-6-desaturase. Excess in one family of fatty acid can interfere with the metabolism of the other and alter their overall biological effects [5]. During n-6 PUFA conversion, delta-6-desaturase, or fatty acid desaturase 2 (FADS2), converts LA to gamma-linolenic acid (GLA, 18:3 n-6). This enzyme represents a rate-limiting step in the synthesis of AA from LA [6]. GLA is elongated to dihomo-gamma-linolenic acid (DGLA, 20:3 n-6) through a chain reaction of four enzymes: a condensation reaction of the fatty acyl chain with malonyl-CoA, catalyzed by an enzyme encoded by the ELOVL5 gene (elongation of very long-chain fatty acids, family member 5); a reduction reaction mediated by 3-ketoacyl-CoA reductase (KAR); a dehydration reaction catalyzed by 3-hydroxyacyl-CoA dehydratase (HACD); and a second reduction reaction catalyzed by trans-2,3-enoyl-CoA reductase (TECR). Finally, DGLA is converted to AA by delta-5 desaturase, or fatty acid desaturase 1 (FADS1) [6, 7]. Interestingly, malonyl-CoA, which is necessary for fatty acid elongation, is derived from the rate-limiting enzyme of the *de novo* fatty acid synthesis pathway, acetyl-CoA carboxylase. Fatty acid synthesis is well described as an overactive pathway in many cancers [8–11], and its upregulation may also contribute to the elongation of PUFA. 

 In contrast to AA, the efficiency of ALA conversion to DHA appears to be very low, below 5% in humans. Most ingested ALA is subject to beta-oxidation to provide energy, and only a small fraction is converted to EPA [12, 13]. It was estimated that as low as 0.2% of ALA is converted to EPA, 64% of EPA to docosapentaenoic acid (DPA, 22:5 n-3), and 37% of DPA to DHA [14]. Thus, the overall amount of DPA and DHA converted from ALA is about 0.13% and 0.05% of the starting ALA, respectively. These findings suggest that any contributions from the fatty acid synthesis pathway toward PUFA metabolism most likely favor n-6, rather than n-3, PUFA elongation. It is also very likely that synthesis of the longer n-3 fatty acids from ALA within the body is competitively hindered by the n-6 analogues. It has been reported that the n-3 conversion efficiency is greater in women, possibly because of the importance of meeting the DHA demands of the fetus and neonate [14].

## 3. PUFA and Cancer

 Total fat intake and the ratio of n-6 to n-3 PUFA in the Western diet have increased significantly since the Industrial Revolution [15, 16]. Increased fat consumption has been associated with the development of specific types of cancer such as breast, colon, and pancreatic and prostate cancers, with the notable exception of n-3 PUFA, which show protective effects against colon, breast, and prostate cancers in a number of experimental systems [17–23]. Epidemiological studies about the association of dietary fat and cancer suggests a protective effect of n-3 PUFA and a promoting effect of n-6 PUFA on cancer. Most clinical data regarding the effects of dietary fat on cancers are observational [24], and the results of such studies are mixed, as many fail to demonstrate a significant association between n-3 PUFA and reduced prostate cancer risk or tumor growth [20, 25–27].

 The Western diet contains disproportionally high amounts of n-6 PUFA and low amounts of n-3 PUFA, denoted as a high n-6 to n-3 PUFA ratio. Most data regarding the effects of high dietary n-6 PUFA are positively associated with prostate cancer incidence [28–30]. In a study of Jamaican men undergoing prostate biopsy for elevated PSA levels, a positive correlation was observed between n-6 fatty acid LA and Gleason score and n-6 (LA) to n-3 (DHA) ratio in erythrocyte membranes and prostate tumor volume [31]. By comparing PUFA content from malignant and benign prostatic tissues from the same prostate specimens, a Swedish research group found that n-6 PUFA and n-6 PUFA precursors were significantly higher in malignant tissues. This finding further demonstrates that n-6 dietary fat is associated with prostate carcinogenesis [28]. In race-specific analyses based on a case-control study comprising 79 prostate cancer cases and 187 controls, Williams and colleagues found that a high ratio of n-6 to n-3 fatty acids may increase the overall risk of prostate cancer among white men and possibly increase the risk of high-grade prostate cancer among all men [29].

 At the same time, epidemiological literature on the association of n-3 PUFA and cancer, including correlational studies and migrational studies, suggest a protective role played by n-3 PUFA. In a recent population-based prospective cohort study of 90,296 Japanese subjects, Sawada et al. reported that consumption of n-3-rich fish or n-3 PUFA, particularly EPA, DPA, and DHA, appears to protect against the development of hepatocellular carcinoma (HCC) [32]. In another population-based prospective study in Japan, there was an inverse relationship between marine n-3 PUFA intake and the risk of colorectal cancer, but this association was only statistically significant in the proximal site of the large bowel [33]. Chavarro et al. performed a nested case-control study by analyzing blood samples of 14,916 healthy men and concluded that higher blood levels of long-chain n-3 fatty acids were associated with a reduced risk of prostate cancer [34]. Szymanski et al. conducted a meta-analysis of fish intake and prostate cancer by focusing on the incidence of prostate cancer and prostate cancer-specific mortality. Their results did not establish a protective association of fish consumption with prostate cancer incidence but showed a significant 63% reduction in prostate cancer-specific mortality [35].

 The results of correlational studies are mixed, some of them failing to demonstrate a statistically significant effect. Several confounding factors could account for the inconsistent results on the association between n-3 PUFA and prostate cancer. First, population-based studies mainly rely on data from self-reported dietary fatty acid intake or from estimates based on national consumption, and these assessments correlate poorly with direct measurements of fatty acids in patient samples. In addition, the actual intake in n-3 PUFA may be too low for a protective effect in some cases. Second, the ratio of n-6 to n-3 fatty acids may be more important than the absolute amount of n-3 PUFA, as suggested by animal and human studies [16, 36]. Using a prostate-specific Pten knockout mouse prostate cancer model, we showed that a ratio of n-6 to n-3 below 5 was effective in slowing cancer progression [3]. Brown et al. reported that AA might potentiate the risk of metastatic prostate cancer cell migration and seeding at the secondary site *in vivo*, and lowering the n-6/n-3 ratio in diet by uptake of n-3 PUFA might reduce this risk [37].

## 4. Mechanisms of Action

### 4.1. Integration of PUFAs into Plasma Membrane Glycerophospholipids

 Although fatty acids are consumed at high levels in a typical Western diet, tumor cells display a strong dependence on *de novo* fatty acid synthesis [9, 10]. The increased proliferation and metabolism of cancer cells could be the trigger for the abnormal requirement for fatty acid compared to normal cells. Most newly synthesized fatty acids are used to support membrane biogenesis in the form of glycerophospholipids, a class of lipids that are a major component of all cell membranes. Glycerophospholipids, including phosphatidylcholine (PC), phosphatidylserine (PS), phosphatidylethanolamine (PE), and phosphatidylinositol (PI), contain a diglyceride, a phosphate group, and a simple organic molecule, such as choline or serine.

 Dietary PUFA can influence the fatty acid composition of glycerophospholipids in cell membranes. In mammals, the *sn*-1 position on the glycerol backbone of glycerophospholipids is usually linked to a saturated fatty acid such as stearic acid (SA, 18:0), and the *sn*-2 position is linked to an n-6 PUFA, such as AA. Feeding cells in culture, or animals, with n-3 PUFA can replace n-6 with n-3 fatty acid at the *sn*-2 position of glycerophospholipids; this is considered a diet-induced *sn*-2 fatty acid moiety change [17, 38, 39]. We have analyzed the incorporation efficiency of PUFAs into glycerophospholipids in prostate cancer cells. Approximately 25% of input albumin-conjugated fatty acids were incorporated into cells within 48 hours. The majority of these newly integrated PUFAs were in the form of PC and PE [40]. These data clearly suggest that the fatty acid at the *sn*-2 position of glycerophospholipids is influenced by cellular PUFA uptake.

 n-3 PUFA influences cell membrane conformation and signaling dynamics. The greater density of n-3 PUFA, compared to n-6 PUFA, dictates their aggregation near the lipid-water interface. This characteristic can significantly affect plasma membrane properties, including membrane fluidity, phase behavior, and permeability [41]. These membrane perturbations can bring about changes associated with receptor activation, such as diffusional coupling of various peripheral proteins required in G protein-coupled receptor signaling (GPCR) [42, 43]. Additionally, because of its high level of unsaturation, DHA has very poor affinity for cholesterol, which is enriched in lipid rafts of the cell membrane. Lipid rafts are important membrane domains for cell signaling as many receptors and proteins are enriched in this domain, such as epidermal growth factor receptor (EGFR) [44]. Hence, incorporation of n-3 PUFA into membrane lipids can disturb the formation of lipid rafts and suppress raft-associated cell signal transduction [13, 44].

 The serine/threonine protein kinase AKT (protein kinase B) is activated in many solid tumors and hematological malignancies. AKT acts downstream of phosphatidylinositol 3-kinase (PI3K) signaling and is a key regulator of multiple survival pathways. AKT is known to phosphorylate and inactivate the proapoptotic Bcl-2 family member BAD as one of its prosurvival tactics [45]. Phosphatidylinositol (PI) is a negatively charged constituent of lipid membranes. Specific kinases phosphorylate the hydroxyl groups on positions 3′, 4′, or 5′ of the inositol ring, and PI3K primarily generates PI-3,4,5-trisphosphate (PIP_3_) from PI-4,5-bisphosphate (PI(4,5)P_2_). PIP_3_ acts as a second messenger to activate pleckstrin homology (PH) domain-containing proteins, including AKT. Conversely, PIP_3_ is hydrolyzed to PI(4,5)P_2_ by PTEN, opposing the action of PI3K [46, 47]. AKT can also be phosphorylated and activated by phosphoinositide-dependent kinase-1 (PDPK1), a PH domain-containing kinase downstream of PI3K (for review, see Franke, 2008) [48]. Hence, intracellular PIP_3_ plays a pivotal role in this PI3K/PIP_3_/AkT cascade pathway.

 Using prostate-specific Pten knockout mice, an immune-competent, orthotopic prostate cancer model, and diets with defined PUFA levels, we found that n-3 fatty acid reduced prostate tumor growth, slowed histopathological progression, and increased survival, whereas n-6 fatty acid had opposite effects. Introducing an n-3 desaturase, which converts n-6 to n-3 fatty acid, into the Pten knockout mice fed an n-6 diet reduced tumor growth similarly to mice fed the n-3 diet. Tumors from mice on the n-3 diet had lower proportions of phosphorylated BAD and higher apoptotic indexes compared with tumors from mice on the n-6 diet. These data suggest that n-3 PUFA can promote BAD-dependent apoptosis to modulate prostate cancer development [17]. We also found that PUFAs modify glycerophospholipid content. DHA can replace the fatty acid at the *sn*-2 position of the glycerol backbone, thereby changing the species of phospholipid. DHA also inhibited AKT^T308^ but not AKT^S473^ phosphorylation, altered PIP_3_ and phospho-AKT^S473^ protein localization, decreased pPDPK1^S241^-AKT and AKT-BAD interaction, and suppressed prostate tumor growth. Knockdown of BAD eliminated n-3 PUFA-induced cell death, and reintroduction of BAD restored the sensitivity to n-3 fatty acids *in vitro*. Knockout of BAD diminished the suppressive effect of n-3 PUFA on prostate tumor growth *in vivo*. These data suggest that modulation of prostate cancer development by PUFA is mediated in part through the PI3K/AKT survival pathway [17, 40]. 

Hu et al. reported that n-3 PUFA-induced apoptosis in human prostate cancer cells occurs through upregulation of syndecan-1 (SDC-1) expression followed by concomitant suppression of PDPK1/AKT/BAD phosphorylation [49]. n-3 fatty acids may also decrease cell proliferation and induce apoptotic cell death in human cancer cells by decreasing signal transduction through the AKT/NF*κ*B cell survival pathway and by modulating the PI3K/AKT/p38 MAPK pathway [50, 51].

### 4.2. PUFA Mediator

 A common fate of unsaturated lipids released from the membrane is oxidation. AA is the precursor of highly bioactive lipid mediators metabolized by a number of enzymes belonging to the cyclooxygenase (COX) and lipoxygenase (LOX) families, as well as cytochrome P450. COXs have two well-characterized isoforms, COX1 and COX2. COX1 is a constitutively expressed gene in most tissues, whereas COX2 is an immediate-early response gene, which is strongly induced in many human malignancies [52]. Signal transduction of n-6 PUFA-derived lipids and the effect of these lipid mediators on the organism have been well characterized. For example, AA-derived lipid mediators are associated with a variety of activities, including inflammation and cancer. Evidence from human studies also supports the important role of COXs and LOXs in PUFA metabolism and cancer [53–56]. Because of its high expression in inflammation and cancer, COX2 has been the subject of intense study and proposed as a target for cancer therapy [57–59].

 In contrast to n-6 PUFA, the metabolism of n-3 PUFA is not well understood. Interest in n-3 PUFA-derived lipid mediators began with observations of Greenland Eskimos whose diet is rich in marine-derived fish and showed lower mortality from coronary heart disease and lower prevalence of inflammation-related diseases, such as psoriasis, inflammatory bowel disease, asthma, rheumatoid arthritis, and other autoimmune diseases [60, 61]. Serhan et al. reported that inflammatory exudates in the murine air pouch from mice treated with n-3 PUFA and aspirin contained a series of bioactive compounds. Using an unbiased lipidomics approach, they identified and named the EPA-derived E-resolvins (RvE1 and RvE2), DHA-derived D-resolvins (RvD1 and RvD2), and (neuro-)protectin (PD1) [62].

RvE1 and RvE2 are protective in a wide variety of disease models, mainly through their anti-inflammatory activities. RvE1 can resolve inflammation caused by bacterial infection of periodontal disease in a rabbit model [63], prevent neovascularization after oxygen-induced retinopathy [64], and suppress neutrophil infiltration in an acute peritonitis model [65]. RvD1, RvD2, and PD1 also have protective activities in a variety of animal models, including models of lung injury, insulin resistance, peritonitis, wound healing, and atherosclerosis [66]. RvD1 was shown to reduce leukocyte infiltration in murine inflammatory exudates [67] and RvD2 was shown to reverse inflammatory pain in mice [68]. PD1 has been reported to regulate amyloid beta secretion and thereby improve neuronal survival in a mouse model of Alzheimer's disease [69]. Although ample data indicate that these n-3 PUFA-derived mediators can resolve inflammation, little is known about their role in inflammation-related cancers, such as prostate and colon cancers.

Due to the existence of multiple oxygenases, the role of each enzyme in the development of prostate cancer has not been studied systematically in a single system or animal model. Furthermore, studies performed in animals rarely take diet into account. To systematically assess the interaction between oxygenases and dietary PUFA in a single animal model of prostate cancer, we knocked out Cox1, Cox2, Lox5, Lox12, or Lox15 in prostate-specific Pten null mice. Our preliminary results indicate that tumor growth was significantly increased in Cox1^−/−^ Pten null mice on n-3 diet compared to Cox1-wild-type Pten null littermates. This result suggests that Cox1 is required for the protective effects of n-3 PUFA. Interestingly, tumor growth was decreased in n-6 PUFA fed Cox1^−/−^ Pten-null mice compared to n-6 fed Cox1-wildtype Pten-null mice, suggesting that n-6 metabolites of Cox1 promote tumor growth. Loss of Cox2 reduced prostate tumor growth on both n-3 and n-6 diets, suggesting that the suppressive effect of n-3 PUFA is independent of Cox2 metabolism. Loss of Lox5 reduced prostate tumor growth on n-6 diet but had no effect on n-3 diet; loss of Lox12 or Lox15 did not affect prostate tumor growth on either diet. These results suggest that the promotion of prostate tumor growth by n-6 diet is dependent on Lox-5 metabolism and both Lox12 and Lox15 metabolites are not critical for prostate cancer growth in this model (Chen et al., unpublished).

### 4.3. Fatty Acids Receptors

 Lipids are ligands for cell-surface G protein-coupled receptors (GPCRs), toll-like receptors (TLRs), and peroxisome proliferator-activated receptors (PPARs). G protein-coupled receptors (GPCRs) are important signaling molecules for many aspects of cellular function. They are members of a large family that share common structural motifs, such as seven transmembrane helices and the ability to activate heterotrimeric G proteins. Recently, several groups reported that unbound free fatty acids can activate GPCRs, including GPR40, GPR41, GPR43, GPR84, and GPR120 [3]. Short-chain fatty acids are specific ligands for GPR41 and GPR43, medium-chain fatty acids for GPR84, and long-chain fatty acids for GPR40 and GPR120 [70–73]. Activation of GPR84 receptor by medium-chain fatty acids triggered the production of the proinflammatory cytokines from leukocytes and macrophages. The function of GPR84 may be associated with chronic low-grade inflammation-associated disease [74].

 GPR40 and GPR120 have been reported to be activated by long-chain fatty acids such as DHA, EPA, and AA [73, 75]. As a G protein-coupled receptor, GPR40 can activate the phospholipase C and phosphatidylinositol signaling pathways [76]. Although GPR40 is preferentially expressed in pancreatic *β*-cells and is known to mediate insulin secretion [77], several groups showed that it is expressed in the brain where it mediates the antinociceptive activity of DHA [78, 79]. Recently, Oh and others reported that GPR120 functions as an n-3 PUFA receptor *in vitro* and *in vivo* [3] and suggested that diminished activation of GPR120 can be an important contributor to obesity, insulin resistance, and tissue inflammation [80, 81]. GPR120 is highly expressed in adipose tissue, proinflammatory bone marrow-derived CD11C^+^ macrophages (BMDCs), mature adipocytes, and monocytic RAW 264.7 macrophage cells. DHA strongly inhibited LPS-induced phosphorylation of JNK and IKK*β*, I*κ*B degradation, cytokine secretion- and inflammatory gene expression level in GPR120-positive cells. These effects of DHA were completely prevented by GPR120 knockdown, demonstrating that these anti-inflammatory effects were specifically exerted through GPR120. An n-3 PUFA diet containing 27% fish oil led to improved insulin sensitivity with increased glucose infusion rates, enhanced muscle insulin sensitivity, and greater hepatic insulin sensitivity. The n-3 PUFA diet had no effect in the GPR120 knockout (KO) mice. On chow diets, the GPR120 KO mice showed moderate insulin resistance with no changes in food intake or body weight. On high-fat diet (HFD), the GPR120 KO mice gained more weight than wild-type controls [3]. In humans, GPR120 expression in adipose tissue is significantly higher in obese individuals than in lean controls [80]. Ichimura and colleagues compared sequences of GPR120 exons in obese populations and discovered a deleterious nonsynonymous mutation (R270H) [80]. Their population study showed that the GPR120^R270H^ variant correlated with obesity. Further investigation *in vitro* demonstrated that the GPR120^R270H^ variant was unable to respond to long-chain fatty acid stimulation. This inactive mutant of GPR120 may contribute to its significant association with obesity [80].

 Toll-like receptors (TLRs) are transmembrane glycoprotein receptors that are important regulators of the innate immune system. TLRs are considered a link between innate (nonspecific) and adaptive (specific) immunity and contribute to the immune system's capacity to efficiently combat pathogens [82]. TLR expression is increased in tumors, including breast, colorectal, melanoma, lung, prostate, pancreatic, and liver cancer [83]. Activation of TLRs triggers a signaling cascade producing inflammatory cytokines that recruit components of the adaptive immune system to kill the pathogen [84]. Among the family of TLRs, TLR4 and TLR9 have been reported to be associated with prostate cancer [85–87]. Panigrahy et al. reported that saturated fatty acids activated, and DHA inhibited, TLR2- and TLR4-mediated proinflammatory activity in a cell culture system [88]. Saturated fatty acids may stimulate the TLR4 signaling pathway to trigger the production of proinflammatory mediators, which may contribute to neuronal death [89]. 

 PPARs (PPAR*α*, PPAR*β*/*δ*, and PPAR*γ*) are a superfamily of ligand-activated transcription factors and nuclear hormone receptors. The syndecan family of cell surface proteoglycans share a structure of small, conserved cytoplasmic and transmembrane domains and larger, distinct ectodomain. They are implicated in a variety of physiologic and pathologic processes such as nutrient metabolism, energy homeostasis, inflammation, and cancer. Growing evidence has demonstrated that PPAR*γ* serves as a tumor suppressor in cancer (see review of Robbins and Nie, 2012) [90]. n-3 PUFA can induce apoptosis in human prostate cancer cells by activating the nuclear receptor PPAR*γ* and upregulating the PPAR*γ* target gene, syndecan-1 (SDC-1) [49, 91]. It has been suggested that n-3 PUFA induces cell apoptosis in prostate cancer via a mechanism of LOX15-mediated SDC-1-dependent suppression of PDPK1/AKT/BAD phosphorylation [49, 91]. SDC-1 upregulation by DHA has also been demonstrated in human breast cancer cells [19, 92–94] and in n-3 PUFA-enriched mammary glands and liver of fat-1 mice [95].

 SDC-1 plays several important cellular functions. It regulates many steps of leukocyte recruitment in noninfectious inflammatory diseases, attenuates inflammation by modulating heparin sulfate-binding proinflammatory factors, and plays a key role in the normal remodeling of injured cardiac tissues [96]. Loss of cell surface SDC-1, seen in many carcinomas such as skin cancer and colorectal adenocarcinomas, favors acquisition of the metastatic phenotype in cancer cells [97]. There is very limited information about SDC-1 expression in prostate cancer. Some studies reported an inverse relationship between SDC-1 and Gleason score [98, 99], but a tissue microarray analysis in another study showed an increase in SDC-1 with tumor progression [100]. Our own studies have shown reduced expression of SDC-1 in prostate cancer cell lines compared to normal prostate epithelial cells and lower expression in androgen-dependent LNCaP cells compared to androgen-independent PC3 and DU145 cells [49]. In a mouse prostate cancer model, the reduction in tumor growth as a result of dietary n-3 PUFA is accompanied by an increase in the expression of secreted SDC-1 [49, 101].

 Several other receptors have been suggested as targets for n-3 PUFA action. Turk et al. reported that DHA can induce the alteration in both the lateral and subcellular localization of EGFR and suppress EGFR signaling, which suggests implications for the molecular basis of cancer prevention by DHA [44, 102]. It is also reported that DHA can increase CD95 (Fas ligand death receptor) cell surface expression and may mediate CD95-induced apoptosis [103]. For more information about PUFA receptor interaction, please refer to a review by Lee et al. [104].

### 4.4. Other Mechanisms

 Nuclear factor erythroid-2-related factor 2 (Nrf2) is a basic leucine zipper transcription factor. Nrf2 is sequestered in the cytoplasm by Kelch-like ECH-associated protein 1 (Keap1) under basal conditions. When the cell is challenged by oxidative stress, Nrf2 is released from Keap1 inhibition, translocates to the nucleus, forms a complex with other factors, and activates transcription of genes containing an antioxidant response element (ARE) in their promoter region. Nrf2 has been reported to play an important role in lung injury reversal, human endothelial cell survival, neuroinflammation, hyperoxia, lung damage from cigarette smoking, and impaired function of macrophages [105]. Other studies suggest that Nrf2 suppresses inflammation by inhibiting NF*κ*B activation through regulation of redox balance, calcium signaling, and PPARs [106]. Various human cancers, such as lung cancer, frequently exhibit increased levels of Nrf2 [107]. Downregulation of nuclear Nrf2 gene expression by RNAi-mediated silencing in nonsmall cell lung cancer inhibits tumor growth and increases efficacy of chemotherapy [108]. Cancer cells are suspected to hijack the Keap1-Nrf2 system as a means to acquire malignant properties. Indeed, the prognosis of patients carrying Nrf2-positve cancers is poor [105]. Oxidized n-3 fatty acids can react directly with the negative regulator of Nrf2, Keap1, by dissociating them and inducing Nrf2-directed gene expression [109]. For example, n-3 PUFA mediators can activate Nrf2 in vascular endothelial cells to prevent oxidative stress-induced cytotoxicity [110]. DHA and EPA can induce Nrf2 expression and suppress lipopolysaccharide-(LPS-) induced inflammation [111]. Evidence of Nrf2-mediated response modulated by n-3 PUFA in prostate cancer came from a randomized clinical trial. Eighty-four men with low-risk prostate cancer were stratified based on self-reported dietary consumption of fish oil. Exploratory pathway analyses of rank-ordered genes revealed the modulation of Nrf2 or Nrf2-mediated oxidative response after 3 months of fish oil supplementation (*P* = 0.01) [112]. 

 Calcium (Ca^2+^) signaling is a ubiquitous mechanism in the control of cell function. The transient receptor potential channels (TRP channels) are 6 transmembrane-spanning proteins with both amino and carboxyl tails located on the intracellular side of the membrane. Ca^2+^ flux through TRP channels located in the plasma membrane and in the membranes of excitable intracellular organelles can promote changes in intracellular free Ca^2+^ concentrations and the membrane potential, which can modulate the driving force for other ions and Ca^2+^ itself [113]. Evidence suggests that TRP channel function can be modulated both directly and indirectly by n-3 fatty acids [114]. It was demonstrated that DHA and EPA at physiological concentrations have the ability to evoke small currents, which seems to be dependent on the previous sensitization of the channel by protein kinase C (PKC). Whether these effects are due to the action of these PUFAs on the agonist binding site or are due to conformational changes caused by TRP protein interactions with the lipid bilayer requires further investigation [115]. Recent findings also indicate that TRP channel function can be modulated by D and E resolvins. Resolvin binding on GPCRs seems to be part of the mechanism underlying the resolvin-mediated regulation of TRP channel function [116]. DHA significantly reduces oxidative stress-induced endothelial cell Ca^2+^ influx. This effect might be associated, at least in part, with altered lipid composition in membrane caveolar rafts [117]. Ca^2+^ has been shown to be essential for increased cell proliferation in prostate cells [118]. Sun et al. observed significantly higher Ca^2+^ influx in prostate cancer cells. They reported that high ratio of Ca^2+^/Mg^2+^ facilitated Ca^2+^ influx and led to a significant increase in cell proliferation of prostate cancer [119]. Thus, one could speculate that n-3 PUFA might indirectly modulate prostate cancer growth by directly modulating Ca^2+^ influx.

## 5. Conclusions

 Cancer incidence and mortality are high in the Western world and a high n-6 to n-3 PUFA ratio in the Western diet may be a contributing factor. There is much evidence to suggest that n-3 PUFA has antiproliferative effects in cancer cell lines, animal models, and humans. Direct effects on cancer cells and indirect effects on the host immune system (anti-inflammation) likely contribute to the inhibitory effect of n-3 fatty acids on tumor growth; however, further investigation is warranted. n-3 PUFA may also regulate other complex metabolic processes, including *β*-oxidation, lipid release from glycerophospholipids, cellular signaling of membrane bound proteins, eicosanoid synthesis, and direct activation of nuclear receptors and gene transcription, all of which may influence the development and progression of prostate cancer. Overall, there seems to be an exceptionally broad potential for the mechanisms mediating cancer prevention by n-3 PUFA (summarized in Table 1). We expect that new research in lipidomics and metabolomics will provide new techniques and approaches to answering the many questions that remain regarding the mechanisms underlying the health benefits of n-3 PUFA.

## Figures and Tables

**Table 1:** Mechanism of n-3 PUFA action.

Target	Mechanism of action	References
Membrane PIPs	Suppress PI3K/AKT/BAD survival pathway	[17, 40]
SDC-1	Suppress PI3K/AKT/BAD and AKT/NF*κ*B survival pathway	[49]
Unknown	Suppress AKT/NF*κ*B or PI3K/AKT/p38 MAPK pathway	[50, 51]
GPR40	Mediate insulin secretion, cell proliferation	[75–77]
GPR120	Anti-inflammation	[3, 80]
TLRs	Suppress the production of proinflammatory mediators	[88, 89]
PPARg	Induce cell apoptosis	[90]
Nrf2	Suppress inflammation, reduce oxidative stress	[106, 109, 110]
Calcium signaling	Inhibit cell proliferation	[114]
COXs	Promote inflammation	[52]
LOXs	Promote inflammation	[53]
E-resolvins	Resolve inflammation	[62]
D-resolvins	Resolve inflammation	[62]

## Abbreviations

PUFA: Polyunsaturated fatty acid
EPA: Eicosapentaenoic acid (20:5, n-3)
DHA: Docosahexaenoic acid (22:6, n-3)
LA: Linoleic acid (18:2, n-6)
AA: Arachidonic acid (20:4, n-6)
ALA: Alpha linolenic acid (18:3, n-3)
FADS: Fatty acid desaturase
GLA: Gamma-linolenic acid (18:3, n-6)
DGLA: Dihomo-gamma-linolenic acid (20:3, n-6)
KAR: 3-Ketoacyl-CoA reductase
HACD: 3-Hydroxyacyl-CoA dehydratase
TECR: Trans-2,3-enoyl-CoA reductase
DPA: Docosapentaenoic acid (22:5, n-3)
PC: Phosphatidylcholine
PS: Phosphatidylserine
PE: Phosphatidylethanolamine
PI: Phosphatidylinositol
SA: Stearic acid (18:0)
GPCR: G Protein-coupled receptor
EGFR: Epidermal growth factor receptor
AKT: Serine/threonine protein kinase (protein kinase B)
PI3K: Phosphatidylinositol-3-kinase
PIP_3_: PI-3,4,5-trisphosphate
PH: Pleckstrin homology
PDPK1: Phosphoinositide-dependent kinase-1
SDC-1: Syndecan-1
COX: Cyclooxygenase
LOX: Lipoxygenase
RvE1: E-Resolvin1
RvD1: D-Resolvin1
TLR: Toll-like receptor
PPAR: Peroxisome proliferator-activated receptor
MCFA: Medium-chain fatty acid
BMDC: Bone marrow-derived CD11C^+^ macrophage
KO: Knockout
HFD: High-fat diet
Nrf2: Nuclear factor erythroid-2-related factor 2
Keap1: Kelch-like ECH-associated protein 1
ARE: Antioxidant response element
LPS: Lipopolysaccharide
PKC: Protein kinase C.
